# *Salix* transect of Europe: latitudinal patterns in willow diversity from Greece to arctic Norway

**DOI:** 10.3897/BDJ.3.e6258

**Published:** 2015-10-30

**Authors:** Quentin Cronk, Enrico Ruzzier, Irina Belyaeva, Diana Percy

**Affiliations:** ‡ Department of Botany, University of British Columbia, Vancouver, BC V6T 1Z4, Canada; § Department of Life Sciences, Natural History Museum, London SW7 5BD, United Kingdom; | Department of Bioinformatics and Spatial Analysis, Royal Botanic Gardens, Kew, Richmond, Surrey TW9 3AB, United Kingdom

**Keywords:** Biogeography, Bulgaria, ecospace, Estonia, Finland, Greece, Hungary, latitudinal gradient, Latvia, Lithuania, megatransect, Norway, Poland, Romania, Salicaceae, salicophagy, spatial analysis, willow-feeding insects

## Abstract

**Background:**

Willows (*Salix* spp.) are ecosystem "foundation species" that are hosts to large numbers of associated insects. Determining their patterns of distribution across Europe is therefore of interest for understanding the spatial distribution of associated fauna. The aim of this study was to record species composition at multiple sites on a long latitudinal gradient (megatransect) across Europe as a baseline for the future detailed analysis of insect fauna at these sites. In this way we used willow stands as comparable mesocosms in which to study floristic and faunistic changes with latitude across Europe.

**New information:**

To determine spatial patterning of  an ecologically important group on a latitudinal gradient across Europe, we sampled willows at the stand level in 42 sites, approximately 100 km apart, from the Aegean (38.8°N) to the Arctic Ocean (70.6°N), but at a similar longitude (21.2 to 26.1°E). The sites were predominantly lowland (elevations 1 to 556 metres amsl, median = 95 m) and wet (associated with rivers, lakes, drainage ditches or wet meadows). The median number of willow taxa (species and hybrids) per stand was four, and varied from one to nine. There is a progressive increase in willow diversity from south to north with the median number of taxa per stand in southern Europe being three, and in northern Europe six. A total of 20 willow species were recorded, along with 12 hybrids. The most widespread willow in the transect was *Salix alba* L. (occurring in 20 sites out of 42) followed by *S. triandra* L. (15 sites), *S. caprea* L., *S. phylicifolia* L. (14 sites) and *S. myrsinifolia* Salisb., *Salix ×fragilis* L. (13 sites). Voucher specimens from this study are deposited in the herbaria of the Natural History Museum (BM) and the Royal Botanic Gardens Kew (K). These samples provide a "snapshot" of willow diversity along a latitudinal gradient and an indication of the geographically changing taxonomic diversity that is presented to willow-feeding herbivores across Europe. It is anticipated that further papers will examine the insect fauna collected from these sites as part of this study.

## Introduction

### The ecological significance of the genus *Salix*

Willows (the genus *Salix* L.) are "foundation species" (Ellison et al. 2005) in many wet habitats in the north temperate region. By providing an abundant food-source for many willow-feeding animals (generalists and specialists) they provide the basis for characteristic ecosystems (Brändle and Brandl 2001, Nyman et al. 2007). Willow leaves frequently show signs of leaf damage resulting from herbivore feeding. Herbivores include mammals: rodents (Tahvanainen et al. 1985b), deer, elk and, in the arctic, reindeer (den Herder and Niemelä 2003) and also phytophagous insects, notably Lepidoptera, Coleoptera and Symphyta Hymenoptera (sawflies) (Volf et al. 2015).

Phytophagous Coleoptera have, in addition to generalists that may potentially or sporadically feed on willow, several *Salix* specialists (Häggström and Larsson 1995, Volf et al. 2015) that will cue in on willow phytochemistry (Kolehmainen et al. 1995, Rowell-Rahier 1984a, Rowell-Rahier 1984b, Tahvanainen et al. 1985a). The suborder Symphyta contains a number of highly specialised willow-feeders (Leppänen et al. 2014, Roininen and Tahvanainen 1989) and are particularly abundant in Northern Europe, a fact which has been attributed to the greater number of willows in the north (Kouki et al. 1994). Willows are also host to numerous sap-sucking insects in the Hemiptera, especially aphids (Aphididae) (Blackman and Eastop 2014), psyllids (Psyllidae and Triozidae: Hill and Hodkinson 1995, Hill et al. 1998, Hodkinson et al. 2001, Serbina et al. 2015), leaf-hoppers (Cicadellidae) and spittle-bugs (Cercopidae).

The abundant herbivores further support a predator trophic level, from birds (Sipura 1999), ants and predatory beetles, as well as large numbers of parasitic wasps (Callan 1940). The diversity of willow-feeding herbivores suggests that willows can be considered a "superhost". The concept of superhost is usually applied to hosts of galling insects (de Araújo et al. 2013). Willows do indeed host many galling insects, but also act as a superhost more generally for many guilds of herbivorous insects. In a survey of 25 European tree species, willows had both the greatest number of phytophages and the greatest number of specialist herbivores (Brändle and Brandl 2001).

### Taxonomy of willow

The genus *Salix* in Europe is usually considered to be a difficult and confusing group for classification and identification (Karrenberg et al. 2002, Meikle 1992, Rechinger 1992, Skvortsov 1999). The main reasons for this are: (1) genetically-based morphological polymorphism, (2) phenotypic plasticity (3) the prevalence of hybridization (4) differences in taxonomic opinion. Although some willows (such as *S. pentandra* L.) are rather uniform, other species are highly variable. *Salix myrsinifolia* is a good example of a species that shows extensive polymorphism: notably in leaf indumentum (hairy to glabrous) and leaf shape (narrowly to broadly elliptical). Although willow polymorphism is rarely formally tested in common garden experiments it is likely that much of this polymorphism is genetically based as different morphs can be found mixed in populations, having developed under the same environmental conditions.

Willows also exhibit phenotypic plasticity, such that even different plants of the same clone can look quite different, particularly if coppiced. Coppice shoots and their leaves can be very different from those of normal branches. However, probably the most remarkable and problematic aspect of willow taxonomy is the great ability for willows to hybridize. Crosses between quite unrelated species occur and many hybrids have a high degree of fertility. A recent study has shown that widespread hybridization is sufficient for chloroplast capture to occur even when species boundaries are maintained (Percy et al. 2014).

Coupled with this is the fact that many hybrids are of economic importance, due to their fast growth, and are widely planted. An example is the widespread hybrid *Salix ×rubra* Huds. (*S. purpurea* L. × *S. viminalis* L.). Another case is *S. ×meyeriana* Rostk. ex Willd. (*S. euxina* I.V.Belyaeva × *S. pentandra*) frequently planted as a more easily propagatable alternative to *S. pentandra* (Zinovjev 2011). Sometimes hybrids are so widespread they behave effectively as homoploid hybrid species. An example is the crack willow (*S. ×fragilis*) which is a hybrid between *S. alba* and *S. euxina* (Belyaeva 2009) but which constitutes a characteristic landscape feature over much of Europe and which authors have in the past considered a species (Meikle 1984). Another case where taxonomic treatment varies is *S. bebbiana* Sarg. Here, we follow Skvortsov (Skvortsov 1999) in recognizing *S. bebbiana* as an Eurasian as well as a North American species, despite considerable variation across the range. However, many European authors (e.g. Bennett et al. 1991, Rechinger and Akeroyd 1993) consider the European *S. bebbiana* to represent a separate species, *S. xerophila* Flod. *Salix bebbiana* (=*S. xerophila*) is closely related to the glabrescent Pale Willow (*S. starkeana* Willd.). However, *S. starkeana* is a comparatively rare willow.

### Geography of willow and stand level sampling

Species of the genus *Salix* have a long history of being mapped in Europe starting with the monumental *Atlas Florae Europaeae* project (Jalas and Suominen 1976). In turn, these data have been used for detailed analyses of geographic distribution using numerical methods at a continent-wide (Myklestad and Birks 1993) and regional (Myklestad 1993) scale. A more recent resource at the country level (with more up-to-date taxonomy) is that of the Euro+Med Plantbase (Uotila 2011). However, stand composition cannot be easily predicted from occurrences in large grid squares or whole countries. Willows in natural stands across Europe provide a distinctive ecospace for the willow-feeding organisms and understanding the changing stand-level taxonomic composition of the *Salix* species is important for understanding the host choice and distribution of willow herbivores. It is the stand that provides the landscape unit and the ecospace within which host choice operates. Also large-scale mapping projects often exclude hybrids, which may be an important part of natural stands and particularly important as they may possibly form "hybrid bridges" (Floate and Whitham 1993) for herbivorous insects to move between hosts. Furthermore, direct observation of natural willow stands, as in this study, allows the co-collection of herbivores with the collection of voucher herbarium specimens.

The collection of data over a long geogrphical distance falls into the category of studies now dubbed 'megatransects'. The power and utility of this technique has been amply demonstrated by numerous recent studies. Some recent examples include: Anstett et al. 2014, Baltensperger and Huettmann 2015, Baltensperger et al. 2015, Barrios-Garcia et al. 2014, Hernández et al. 2007, Huber 2015, Senterre et al. 2004.

## Material and methods

### Stand selection

**Figure 1.**
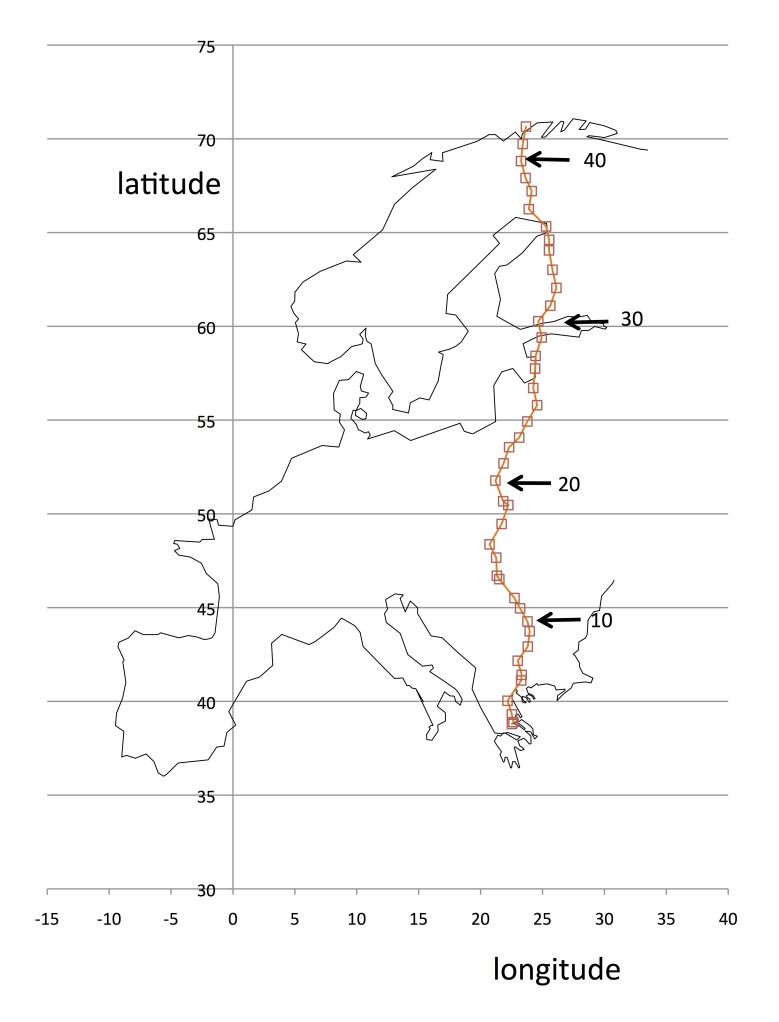
Map of sampling sites with sites 10, 20, 30 and 40 arrowed.

Willow stands were examined during two journeys by road across Europe: Greece to Poland in April 2015 and Poland to Norway in June 2015 (Fig. 1). Sites were selected by driving approximately 100 km north of the previous site and opportunistically locating a suitable habitat in which to find willows, generally a river or low-lying ground. The spacing of sites varied according to logistic and travel constraints. In southern Europe, willows are largely restricted to riparian habitats, but northwards they become commoner in many more habitats. The sampling unit was the willow stand (willow dominated local area). The requirement for a site was that it had a stand of willows that met certain minimum size requirements (at least 100 m in longest linear dimension). A stand of willows is defined as a contiguous area where willows are the dominant vegetation for at least 100 m in linear dimension (as for instance along a river bank). Some stands are very extensive, in which case our sampling was limited to approximately 200 m in largest linear dimension. Because willow stands differ so much in size, shape and density, it was not practical to impose equal area or grid sampling. Time constraints limited entomological and botanical sampling to 1-2 hours per site. A total of 42 sites were sampled across Europe from south to north (38.8 to 70.6°N) while minimising east-west deviation to between approximately 21.2 and 26.1°E. In addition to the 42 sites, a series of "Supplemental sites" are recorded at which additional insect collections were made but the full site recording process was not carried out.

### Data collection

At each site latitude, longitude and altitude data were collected using a hand held Garmin Etrex global positioning system, accurate to within 3 m. Basic notes on the immediate environment were taken to provide a habitat profile of the sites. At each site, the willow diversity was determined and voucher specimens were made in order to validate the species present and to capture variation in species that exhibited considerable phenotypic variation. If the willows were flowering, an attempt was made to collect both male and female individuals. Willow abundance relative to abundance of other woody plants was estimated on a four-point scale: 1) abundant - 30% of individuals or more; 2) common - 10-30%; 3) occasional <10%; 4) rare - one or two individuals only were detected. Samples were processed using standard herbarium techniques and specimens are deposited at the Natural History Museum, London (BM) or at the Royal Botanic Gardens Kew (K). Field identifications were made by QC and DMP. Confirmation, and critical determination of all vouchers, including hybrids, was done by IB. In addition to herbarium samples, samples of leaf tissue were dried using silica gel to permit future DNA-based studies and retained at NHM.

### Climate data

As background information, climate data for three contrasting individual sites is given from publically available data sources (Table 2). These use a dataset of mean historical monthly temperature (°Celsius) and rainfall (mm), computed globally for the period 1990-2009 by the Climatic Research Unit (CRU) of University of East Anglia (UEA) and available through the World Bank Climate Portal (World Bank Group 2015).

## Results

### Sites

Table 1 shows the 42 sites recorded in this study as well as details of further "supplemental sites" where insect collections were made but without the level of sampling accorded to the main sites. The geographical distribution of sites is shown in Fig. 1. The supplemental sites will not be discussed here but their basic details are given, as subsequent papers on the insects sampled along the transect may refer to them. The latitudinal variation provides an enormous variation in climate. Table 2 shows summary climatic statistics for three sites: the most southerly, the most northerly and a central site (Poland).

As can be seen from Table 1, site elevations varied from 1 m to 556 m above mean sea level (amsl), with a median of 95 m. Because the sampling was predominantly in lowlands, the diversity of mountain or upland willows was not captured in this study, nor was it intended to be. Instead we capture the diversity of large stands of willow found in wet low-lying areas, which from an "insect eye view" or "insect chemosensory perspective" represent the largest areas of willow resource in the landscape, generally associated with landscape features such as rivers, lakes, drainage ditches or poorly drained meadows.

### Willows

Table 3 lists the total of 20 willow species that were recorded, together with the 12 hybrids. For each taxon the total number of sites (out of 42) is given. In this transect, the most widespread willow is *Salix alba*, which occurs in 20 sites (out of 42). This species is followed by *S. triandra* (with 15 sites), *S. caprea*, *S. phylicifolia* (with 14 sites each) and by *S. myrsinifolia*, *S. ×fragilis* (with 13 sites).

Site diversity (Table 4) was modest with the overall median number of willow taxa (species and hybrids) per stand being four. However, the stands showed a progressive increase in diversity from south to north with the median number of willow taxa per stand in southern Europe (Greece, Bulgaria, Romania) being three; the median number in central Europe (Hungary, Poland) being five; and the median number in northern Europe (Lithuania, Latvia, Estonia, Finland and Norway) being six.

Three stands (in Greece and Bulgaria) had just one willow taxon and in all cases this was *S. alba*. One stand (site 26 in Latvia) had the maximum recorded number of taxa, nine per stand, while six sites (in Hungary, Poland, Lithuania and Norway) had seven taxa. Finally, Table 5 lists the voucher specimens collected.

## Discussion

These samples provide a "snapshot" of willow diversity along a latitudinal gradient and an indication of the geographically changing taxonomic diversity that is presented to willow-feeding herbivores across Europe. What is particularly noticeable is the role in taxic diversity of hybrids. One third (10 out of 30) of the total taxa recorded were hybrids. This highlights the importance of recording hybrids, which are often inadequately reported in surveys. *Salix* hybrids are notable for their frequency in nature but comparative rarity in the literature on willows.

Also worthy of comment is the general increase in willow diversity from south to north (Table 4). This is the opposite of a common biogeographical pattern that species diversity is higher in warmer regions nearer the tropics, and lower nearer the poles. The genus *Salix* has undergone a major radiation in boreal regions which may go some way towards explaining this inversion of the norm.

Finally, it should be noted that these willows formed the background for a major sampling of insects and it is anticipated that further papers forming part of this study will examine the insect fauna collected.

## Figures and Tables

**Table 1.** Latitude, longitude and general details of collecting sites, *Salix* transect across Europe, April-June 2015.

**SITE**	**Lat** °**N**	**Long** °**E**	**Alt (m asl)**	**date**	**country**	**river/site**	**habitat**
1	38.80007	22.462900	37	21-iv-2015	Greece	River (R.) Asopos, west of Thermopylae	Bank of fast-flowing rocky river partly shaded by *Platanus* and with *Arundo* and *Tamarix* along the stream bank
2	38.902000	22.310150	33	21-iv-2015	Greece	R. Sperchios, near Leianokladi, east of Lamia	Bank of wide and rocky river bed in wide floodplain with *Tamarix* and *Rubus* etc.
3	39.306694	22.528323	177	22-iv-2015	Greece	R. Enipeas east of Farsala	By irrigation pumping station, bank of river flowing through agricultural area (fields of wheat). With *Cercis*, *Populus*, *Rubus* etc.
4	40.032685	22.175437	534	22-iv-2015	Greece	Stream near Kokkinogeia, Thrace	Damp drainage in foothills of the Olympus range with rapidly flowing stream meandering through. Rough grassy terrain with poplar and willow trees.
5	41.113317	23.273893	31	23-iv-2015	Greece	At R. Struma, near Lithotopos	In mud and shallow water at the edge of a broad and muddy irrigation canal through agricultural land with willows, poplar and *Rubus* etc.
6	41.412468	23.318609	90	23-iv-2015	Bulgaria	R. Struma, near Topolnitsa	Bank of river in narrow sandy grazed floodplain between the river and hills.
7	42.165622	22.998141	392	24-iv-2015	Bulgaria	R. Struma, north of Boboshevo	Sandy riverbank with poplar, ash, willow and elm between rocky side of gorge and sandy flat with small church/shrine.
8	42.923989	23.810563	339	24-iv-2015	Bulgaria	R. Kalnitza, near Botevgrad	Sandy banks of small polluted river in construction area with *Rubus*, *Urtica*, *Prunus spinosa* etc.
9	43.739343	23.966755	35	24-iv-2015	Bulgaria	R. Danube, at Oryahovo	River bank by light industrial area at ferry port on the Danube with poplars, tree willows, *Phragmites* etc.
10	44.260343	23.786781	81	25-iv-2015	Romania	R. Jiu, at Podari, near Craiova	Clayey/sandy bank of river with white mulberry, poplar etc
11	44.961981	23.190337	172	25-iv-2015	Romania	R. Jiu, north of Rovinari	Along ditches in middle of ploughed fields with poplars and *Phragmites* etc.
12	45.510676	22.737225	556	26-iv-2015	Romania	Meadow near Paucinesti, Carpathian region	Along ditches and in fields in grazed sedgy meadows in agricultural valley. Many plum trees in blossom.
13	46.518504	21.512839	102	26-iv-2015	Romania	R. Crişul Alb, at Chișineu-Criș	Embanked river through town, grassy slope with thick willow patches.
14	46.700744	21.312680	94	27-iv-2015	Hungary	R. Fekete-Körös, near Gyula	Bank of 20m wide river, grassy bank with willows and nettles etc
15	47.665648	21.261768	91	27-iv-2015	Hungary	Drainage ditches near R. Hortobagy, north-east of Balmazújváros	Broad drainage ditch between road and ploughed field with *Phragmites* etc.
16	48.374291	20.725264	148	28-iv-2015	Hungary	R. Bodva, south of Szendrő	Bank of small river running through landscape of forest and agricultural fields. With poplars, Euonymus europaeus and *Prunus spinosa* etc
17	49.463447	21.697255	385	28-iv-2015	Poland	R. Panna, at Tylawa	Bank of small river (7-8 m wide) with stony to muddy bottom and alders, birches and blackthorns etc
18	50.470234	22.238372	157	29-iv-2015	Poland	Fields north of Rudnik nad Sanem	Agricultural land by E77 highway with old and young *Salix viminalis* plantations. Birch, alder and blackthorn common.
19	50.673994	21.823391	141	29-iv-2015	Poland	R. Łęg, near Gorzyce	Wet meadow near embanked river with *Phragmites* etc.
20	51.775039	21.197100	101	30-iv-2015	Poland	R. Pilica, at Warka	Sandy banks of large river (30m wide), banks managed for angling.
20a	51.775039	21.197100	101	11-vi-2015	Poland	R. Pilica, at Warka	Sandy banks of large river (30m wide), banks managed for angling.
21	52.693980	21.852900	96	12-vi-2015	Poland	R. Bug, near Brok	Rough banks of wide muddy river used for angling with nettle, *Rubus*, *Symphytum* etc.
22	53.554830	22.302990	128	12-vi-2015	Poland	Meadow near R. Biebrza at Wasocz, near Szczuczyn	Wet meadow with *Typha*, *Menyanthes*, *Comarum* etc.
23	54.069430	23.117450	137	13-vi-2015	Poland	R. Czarna Hańcza, near Sejny on road from Suwalki	Sluggish 12m wide river with waterlilies and lined with *Phragmites* and *Alnus*, and adjacent meadow with *Cirsium*, *Dactylorhiza* etc.
24	54.925830	23.774200	28	13-vi-2015	Lithuania	Embankment of River at Kaunas	Dry sandy ridge overlooking wide muddy river
25	55.795570	24.566780	62	13-vi-2015	Lithuania	R. Levuo at Karsakiškis near to Panevėžys	Banks of river with birch and willow thicket
26	56.711410	24.251620	23	14-vi-2015	Latvia	Near R. Misa, between Iecava and Kekana	Scrubby meadow beside farm track
27	57.749630	24.402300	7	14-vi-2015	Latvia	R. Salaca short distance inland from Salacgriva	Rough meadow beside river with *Alnus*, *Acer*, *Prunus* etc
28	58.422570	24.440630	18	15-vi-2015	Estonia	Field near Parnu	Rough pasture beside road, invaded by willows
29	59.402890	24.935770	48	15-vi-2015	Estonia	R. Pirita at Lagedi near Tallinn	Banks of small shallow river with abundant aquatic macrophytes, by suspension footbridge
30	60.272990	24.658430	33	16-vi-2015	Finland	Near Lake Bodom, Espoo, Finland	Along ditches near lake, in agricultural landscape of cereal fields and meadows, and aspen/birch groves
31	61.099650	25.628200	84	16-vi-2015	Finland	Drainage flowing into lake Vesijärvi at Paimela near Lahti	Banks of small muddy river 6-7 m wide, in agricultural landscape with abundant aspen and birch
32	62.049620	26.123690	174	17-vi-2015	Finland	Lake near Toivakka	Forest and lake margin where road crosses end of lake in birch, aspen, pine and spruce forest
33	63.015890	25.804570	139	17-vi-2015	Finland	Near Viitasaari	Along ditches beside forest track at margin of *Pinus*, *Betula* forest
34	64.050740	25.526640	91	17-vi-2015	Finland	R. Pyhäjoki, at Joutenniva, south of Haapavesi	Banks of fast flowing rocky river through agricultural landscape with aspens, birches, alders and willows along banks
35	64.612870	25.538050	58	18-vi-2015	Finland	Tributary of the R. Siikajoki near Mankila	Banks of small river (6m wide) and ditches, in agricultural area, with aspen and birch common
36	65.328350	25.291750	1	18-vi-2015	Finland	R. Iijoki at mouth, near Kestilä, north of Oulu	Banks of very wide river
37	66.249470	23.89450	51	19-vi-2015	Finland	Small river between Kainuunkylä and Väystäjä	Wet scrub and woodland edge (birch and spruce) with abundant Trollius and other northern herbs
38	67.212530	24.126290	160	19-vi-2015	Finland	Near Vaattojärvi	Between two small rivers flowing into lake with wet areas and ditches around birch, pine forest
39	67.911830	23.634110	233	19-vi-2015	Finland	R. Muonion (Muonionjoki) just south of Muonio	Banks of wide (100-200m), rocky, fast-flowing river
40	68.813800	23.266580	374	20-vi-2015	Norway	South of Siebe	In birch scrub on heathy ridge above lakeshore in reindeer management area
41	69.724870	23.405810	289	20-vi-2015	Norway	Shores of Lake Trangdalsvatn	Rocky slope down to clear, gravel bottomed lake, surrounded by birch and willow scrub
42	70.652340	23.665830	67	21-vi-2015	Norway	Jansvannet Lake, Hammerfest	Wet areas and margins of birch wood around lake
SUPPLEMENTARY SITES							
i-A	46.847908	8.631778	455	17-iv-2015	Switzer-land	R. Reuss, near Erstfeld	Gravel-bottomed river near motorway
i-C	39.235768	20.523075	-3	19-iv-2015	Greece	R. Acheron, at Mesopotamo	Drained cultivated fields surrounded by drainage ditches in river delta
i-J	45.447181	22.228965	236	26-iv-2015	Romania	Near Caransebes	Wet area near highway interchange [no willows collected]
i-K	52.302400	5.525235	11	1-v-2015	Nether-lands	Dyke in Flevoland	Near abundant drainage dykes by sea on reclaimed land.
ii-A	56.411000	24.167880	33	13-vi-2015	Lithuania	Near Bavska	Planted *S. alba*
ii-B	56.715700	24.249580	12	14-vi-2015	Latvia	R. Misa	Banks of river
ii-C	59.403880	24.932620	43	15-vi-2015	Estonia	Lagedi	Rough grassland invaded by shrubs near houses
ii-D	65.324430	25.315300	6	18-vi-2015	Finland	Kestilä	Margins of birch wood by road
ii-E	66.229570	23.785480	87	19-vi-2015	Finland	Near Kainuunkylä	Wet ditches by road at edge of birch wood
ii-F	67.934880	23.656410	238	20-vi-2015	Finland	Muonio	By river
ii-G	68.458680	23.425840	346	20-vi-2015	Finland	North of Hetta	Rocky area of birch and pine scrub
ii-H	69.343310	23.601290	317	20-vi-2015	Norway	South of Masi	Birch scrub with juniper
ii-I	69.881290	21.731950	7	22-vi-2015	Norway	West of Badderen	Rocky scrub by fjord
ii-J	69.512520	20.703190	13	22-vi-2015	Norway	Near Birtavarre	Rocky scrub by fjord

**Table 2.** Summary climate variables taken from publically available resources (see Methods) for three contrasting sites on the transect: 1 and 42, the most southerly and most northery sites on map (Fig. 1) together with a middle site, 20, indicated by an arrow on map. Mean monthly temperature (°C) and mean monthly precipitation (mm) are given here. This table is provided as background information on the climatic gradient represented by the megatransect.

	*SITE 1 (nr. Thermopylae: 38.80, 22.46)*	*SITE 20 (Warka: 51.78, 21.20)*	*SITE 42 (Hammerfest: 70.65, 23.67)*
**Temperature,°C**			
Jan	5.31	-0.53	-12.51
Feb	7.01	1.02	-12.58
Mar	9.9	3.58	-10.92
Apr	12.41	8.39	-6.08
May	17.8	14.15	0.21
Jun	22.65	16.25	6.96
Jul	24.99	19.4	10.14
Aug	24.76	18.88	8.73
Sep	20.53	13.02	3.99
Oct	16.32	8.65	-3.07
Nov	10.76	2.82	-9.08
Dec	5.28	-3.33	-11.62
**Precipitation, mm**			
Jan	49.06	25.28	79.46
Feb	28.5	29.7	76.35
Mar	43.45	29.48	73.6
Apr	52.78	34.13	54.42
May	44.53	40.14	33.99
Jun	15.56	65.88	42.68
Jul	24.76	79.65	53.09
Aug	15.41	55.42	55.79
Sep	34.78	53.66	51.47
Oct	42.38	42.62	82.58
Nov	66.34	42.03	75.12
Dec	91.11	27.17	87.93

**Table 3.** *Salix* taxa (species and hybrids) on transect.

**Taxon**	**Number of sites on transect**	**Species or hybrid**	**Hybrid binomial (if available)**
*S. alba* L.	20	sp	-
*S. amplexicaulis* Bory & Chaub.	4	sp	-
*S. aurita* L.	6	sp	-
*S. bebbiana* Sarg.	7	sp	-
*S. caprea* L.	14	sp	-
*S. cinerea* L.	9	sp	-
*S. eleagnos* Scop.	1	sp	-
*S. euxina* I.V.Belyaeva	4	sp	-
*S. glauca* L.	5	sp	-
*S. gmelinii* Pall.	1	sp	-
*S. hastata* L.	5	sp	-
*S. lanata* L.	1	sp	-
*S. lapponum* L.	4	sp	-
*S. myrsinifolia* Salisb.	13	sp	-
*S. pentandra* L.	7	sp	-
*S. phylicifolia* L.	14	sp	-
*S. purpurea* L.	8	sp	-
*S. silesiaca* Willd.	1	sp	-
*S. triandra* L.	15	sp	-
*S. viminalis* L.	9	sp	-
*S. alba* × *S. pentandra*	1	h	*S. ×ehrhartiana* G.Mey
*S. aurita* × *S. myrsinifolia*	1	h	*S. ×coriacea* J.Forbes
*S. cinerea* × *S. aurita*	1	h	*S. ×multinervis* Döll
*S. cinerea* × *S. triandra*	1	h	*S. ×krausei* Andersson
*S. euxina* × *S. pentandra*	1	h	*S. ×meyeriana* Rostk. ex Willd.
*S. myrtilloides* × *S. glauca*	1	h	-
*S. phylicifolia* × *S. myrsinifolia*	2	h	*S. ×tetrapla* Walk.
*S. purpurea* × *S. viminalis*	8	h	*S. ×rubra* Huds.
*S. triandra* × *S. viminalis*	3	h	*S. ×mollissima* Sm.
*S. viminalis* × *S. cinerea*	1	h	*S. ×smithiana* Willd.
*S. alba* × *S. euxina*	13	h	*S. ×fragilis* L.
*S. ×fragilis* × *S. triandra*	1	h	*S. ×alopecuroides* Tausch

**Table 4.** *Salix* collections per site, trans-Europe transect, April-June 2015. Frequency in stands is given in brackets as: A=abundant, C=common, O=occasional, R=rare (see Methods under Data Collection).

**Site**	**Country**	**No. of taxa**	**Willow species and hybrids**
1	Greece	3	*S. alba* (O), *S. eleagnos* (O), *S. purpurea (C)*
2	Greece	3	*S. alba* (C), *S. amplexicaulis* (C), *S. triandra (O)*
3	Greece	1	*S. alba (C)*
4	Greece	3	*S. alba* (C), *S. amplexicaulis* (C), *S. triandra (O)*
5	Greece	2	*S. alba* (C), *S. triandra (O)*
6	Bulgaria	4	*S. alba* (C), *S. amplexicaulis* (C), *S. purpurea* × *S. viminalis* (O), *S. ×fragilis (C)*
7	Bulgaria	4	*S. alba* (C), *S. amplexicaulis* (C), *S. euxina* (O), *S. triandra (C)*
8	Bulgaria	2	*S. alba* (O), *S. euxina (C)*
9	Bulgaria	1	*S. alba (A)*
10	Romania	1	*S. alba (A)*
11	Romania	3	*S. alba* (C), *S. purpurea* × *S. viminalis* (C), *S. triandra* × *S. viminalis (O)*
12	Romania	3	*S. cinerea* (C), *S. silesiaca* (C), *S. ×fragilis (O)*
13	Romania	4	*S. alba* × *S. pentandra* (O), *S. purpurea* (O), *S. triandra* (O), *S. ×fragilis (A)*
14	Hungary	7	*S. alba* (O), *S. euxina* (O), *S. purpurea* × *S. viminalis* (O), *S. triandra* (C), *S. triandra* × *S. viminalis* (C), *S. viminalis* (O), *S. ×fragilis (O)*
15	Hungary	4	*S. alba* (O), *S. cinerea* (C), *S. purpurea* × *S. viminalis (C)*, *S. ×fragilis (C)*
16	Hungary	5	*S. alba* (C), *S. aurita* (O), *S. purpurea* (C), *S. triandra* (C), *S. viminalis (O)*
17	Poland	3	*S. caprea* (R), *S. euxina* (C), *S. purpurea (C)*
18	Poland	5	*S. aurita* (O), *S. cinerea* (C), *S. purpurea* × *S. viminalis* (O), *S. triandra* (O), *S. viminalis (C)*
19	Poland	6	*S. alba* (O), *S. cinerea* (C), *S. purpurea* × *S. viminalis* (C), *S. triandra* × *S. viminalis* (C), *S. viminalis* (C), *S. ×fragilis (C)*
20	Poland	6	*S. alba* (R), *S. gmelinii* (O), *S. purpurea* (C), *S. triandra* (C), *S. viminalis* (O), *S. ×fragilis (A)*
21	Poland	7	*S. alba* (O), *S. cinerea* (R), *S. cinerea × triandra* (R), *S. purpurea* (C), *S. triandra* (A), *S. viminalis* (C), *S. ×fragilis (C)*
22	Poland	2	*S. bebbiana* (A), *S. ×fragilis (C)*
23	Poland	3	*S. bebbiana* (A), *S. myrsinifolia* (R), *S. pentandra (O)*
24	Lithuania	7	*S. alba* (R), *S. caprea* (A), *S. purpurea* (C), *S. triandra* (C), *S. viminalis* (O), *S. viminalis* × *S. cinerea* (O), *S. ×fragilis (O)*
25	Lithuania	7	*S. alba* (O), *S. cinerea* (O), *S. myrsinifolia* (A), *S. pentandra* (C), *S. purpurea* (C), *S. triandra* (O), *S. ×fragilis (C)*
26	Latvia	9	*S. alba* (O), *S. bebbiana* (O), *S. caprea* (O), *S. cinerea* (A), *S. myrsinifolia* (O), *S. pentandra* (C), *S. purpurea* × *S. viminalis* (O), *S. triandra* (O), *S. viminalis (O)*
27	Latvia	5	*S. bebbiana* (O), *S. myrsinifolia* (A), *S. triandra* (O), *S. viminalis* (C), *S. ×fragilis (C)*
28	Estonia	6	*S. caprea* (C), *S. cinerea x aurita* (R), *S. myrsinifolia* (A), *S. phylicifolia* (C), *S. triandra* (O), *S. ×fragilis (O)*
29	Estonia	4	*S. myrsinifolia* (A), *S. phylicifolia* × *S. myrsinifolia* (R), *S. purpurea* × *S. viminalis* (C), *S. ×fragilis* × *S. triandra (R)*
30	Finland	5	*S. aurita* (R), *S. caprea* (R), *S. cinerea* (R), *S. pentandra* (R), *S. phylicifolia (A)*
31	Finland	6	*S. cinerea* (O), *S. euxina* × *S. pentandra* (O), *S. myrsinifolia* (A), *S. pentandra* (O), *S. phylicifolia* (O), *S. phylicifolia* × *S. myrsinifolia (C)*
32	Finland	6	*S. aurita* (C), *S. bebbiana* (O), *S. caprea* (O), *S. myrsinifolia* (A), *S. pentandra* (C), *S. phylicifolia (C)*
33	Finland	4	*S. aurita* (O), *S. caprea* (C), *S. myrsinifolia* (C), *S. phylicifolia (A)*
34	Finland	3	*S. caprea* (O), *S. pentandra* (R), *S. phylicifolia (A)*
35	Finland	4	*S. aurita* (R), *S. caprea* (O), *S. aurita × myrsinifolia* (R), *S. phylicifolia (A)*
36	Finland	2	*S. myrsinifolia* (O), *S. phylicifolia (A)*
37	Finland	4	*S. caprea* (O), *S. hastata* (O), *S. myrsinifolia* (O), *S. phylicifolia (A)*
38	Finland	6	*S. caprea* (R), *S. glauca* (A), *S. hastata* (O), *S. lapponum* (R), *S. myrtilloides* × *S. glauca* (R), *S. phylicifolia (A)*
39	Finland	6	*S. bebbiana* (R), *S. caprea* (C), *S. glauca* (O), *S. hastata* (O), *S. lapponum* (A), *S. phylicifolia (A)*
40	Norway	2	*S. glauca* (O), *S. phylicifolia (A)*
41	Norway	7	*S. bebbiana* (O), *S. caprea* (C), *S. glauca* (C), *S. hastata* (O), *S. lapponum* (C), *S. myrsinifolia* (C), *S. phylicifolia (C)*
42	Norway	7	*S. caprea* (O), *S. glauca* (C), *S. hastata* (C), *S. lanata* (R), *S. lapponum* (C), *S. myrsinifolia* (C), *S. phylicifolia (C)*
SUPPLEMENTAL SITES			
A-i	Switzerland	2	*S. eleagnos*, *S. purpurea* × *S. viminalis*
C-i	Greece	1	*S. alba*
J-i	Romania	2	*S. cinerea* [not collected], *S. ×fragilis* [not collected]
K-i	Netherlands	1	*S. caprea*
C-ii	Estonia	7	*S. bebbiana*, *S. cinerea*, *S. euxina* × *S. pentandra*, *S. myrsinifolia*, *S. phylicifolia*, *S. ×fragilis, S. ×fragilis* × *S. triandra*
D-ii	Finland	3	*S. aurita* × *S. cinerea*, *S. caprea*, *S. myrsinifolia* × *S. phylicifolia*
E-ii	Finland	2	*S. bebbiana*, *S. lapponum*
H-ii	Norway	5	*S. glauca*, *S. hastata*, *S. lapponum*, *S. myrsinifolia*, *S. phylicifolia*
I-ii	Norway	3	*S. caprea*, *S. hastata*, *S. myrsinifolia*

**Table 5.** *Salix* collections (collectors: Quentin Cronk and Diana Percy), trans-Europe transect, April-June 2015. Accession number is collector-site-number (e.g. QCDP-A-1; QCDP-19-2). Sex is recorded as m=male, f=female, v=vegetative, b=in bud.

**Site**	**No.**	**Country**	**Sex**	***Name***	**Notes**
1	1	Greece	f	*S. purpurea*	To 6m
1	2	Greece	m	*S. eleagnos*	To 2m
1	3	Greece	m	*S. cf. alba*	River-side shrubs to 2m
1	4	Greece	f	*S. alba*	River-side shrubs to 2m
2	1	Greece	f	*S. amplexicaulis*	Shrub to 5m, opposite leaves
2	2	Greece	f	*S. triandra*	Pale bracts and stipules
2	3	Greece	f	*S. alba*	Large tree willow to 20m
3	1	Greece	m	*S. cf. alba*	Large tree to 20 m with fissured bark, 2 stamens per flower
3	2	Greece	f	*S. cf. alba*	Female flowers pedicillate, bracts relatively narrow, not very hairy, brown tipped
3	3	Greece	m	*S. cf. alba*	Small tree to 4m, 1 stamen per flower
3	4	Greece	f	*S. cf. alba*	Female flowers sessile, bracts relatively wide, very hairy
4	1a	Greece	m	*S. cf. alba*	Tall tree to 20m with fissured bark
4	1b	Greece	f	*S. cf. alba*	Tall tree to 20m with fissured bark
4	2a	Greece	m	*S. amplexicaulis*	Shrub to 4m
4	2b	Greece	f	*S. amplexicaulis*	Shrub to 4m
4	3	Greece	m	*S. triandra* (var.)	Shrub to 4m
5	1a	Greece	m	*S. alba*	Grey-barked tree to 10m
5	1b	Greece	f	*S. alba*	Grey-barked tree to 10m
5	2a	Greece	m	*S. triandra* (var.)	Small shrub to 4m
5	2b	Greece	f	*S. triandra*	Small shrub to 4m
6	1a	Bulgaria	m	*S. alba*	Tall grey barked tree to 15m
6	1b	Bulgaria	f	*S. alba*	Tall grey barked tree to 15m
6	2	Bulgaria	f	*S. ×fragilis*	Small tree to 6m
6	3	Bulgaria	m	*S. purpurea* × *S. viminalis*	Small shrub with reddish twigs, 2m
6	4	Bulgaria	f	*S. amplexicaulis*	Small shrub 2m
7	1a	Bulgaria	m	*S. amplexicaulis*	
7	1b	Bulgaria	f	*S. amplexicaulis*	
7	2a	Bulgaria	m	*S. triandra*	
7	2b	Bulgaria	f	*S. triandra*	
7	3a	Bulgaria	m	*S. alba*	
7	3b	Bulgaria	f	*S. triandra*	
7	4a	Bulgaria	m	*S. euxina*	
7	4b	Bulgaria	f	*S. euxina*	
8	1a	Bulgaria	m	*S. euxina*	
8	1b	Bulgaria	f	*S. euxina*	
8	2	Bulgaria	m	*S. alba*	
9	1a	Bulgaria	m	*S. alba*	Tall trees to 30m and possibly planted. Similar trees are very common along the banks of the Danube.
9	1b	Bulgaria	f	*S. alba*	Tall trees to 30m and possibly planted. Similar trees are very common along the banks of the Danube.
10	1a	Romania	m	*S. alba*	Large tree to 20m
10	1b	Romania	f	*S. alba*	Large tree to 20m
11	1	Romania	m	*S. alba*	
11	2	Romania	f	*S. purpurea* × *S. viminalis*	
11	3	Romania	f	*S. triandra* × *S. viminalis*	
12	1a	Romania	m	*S. silesiaca*	Shrub, twigs ridged under bark
12	1b	Romania	f	*S. cinerea*	Tree to 10m, twigs ridged under bark
12	2	Romania	m	*S. ×fragilis* (towards *S. euxina*?)	Glabrous tree to 10m
13	1a	Romania	m	*S. ×fragilis*	Small coppiced growth by river
13	1b	Romania	f	*S. alba* × *S. pentandra*	Small coppiced growth by river
13	2	Romania	f	*S. triandra*	
13	3	Romania	f	*S. purpurea*	
14	1a	Hungary	m	*S. triandra* × *S. viminalis*	To 5m
14	1b	Hungary	f	*S. triandra*	To 5m
14	2a	Hungary	m	*S. euxina*	To 8m
14	2b	Hungary	f	*S. ×fragilis*	To 8m
14	3	Hungary	f	*S. viminalis*	To 6m
14	4	Hungary	f	S. purpurea × S. viminalis	To 2m
14	5	Hungary	f	*S. alba*	To 10m
15	1a	Hungary	m	*S. cinerea*	Shrub to 4m with striae
15	1b	Hungary	f	*S. cinerea*	Shrub to 4m with striae
15	2	Hungary	m	*S. purpurea* × *S. viminalis*	
15	3	Hungary	f	*S. ×fragilis*	
15	4a	Hungary	m	*S. alba*	Small tree
15	4b	Hungary	f	*S. alba*	Large tree, branches weeping
16	1	Hungary	m	*S. alba*	
16	2	Hungary	m	*S. triandra*	
16	3	Hungary	f	*S. purpurea*	
16	4	Hungary	v	*S. viminalis*	
16	5	Hungary	f	*S. aurita*	
16	6	Hungary	m	*S. alba*	
17	1a	Poland	m	*S. euxina*	
17	1b	Poland	f	*S. euxina*	
17	2	Poland	f	*S. purpurea*	
17	3	Poland	f	*S. caprea*	
18	1	Poland	f	*S. viminalis*	Young coppice plantation
18	2a	Poland	m	*S. aurita*	Shrub to 4m weakly striate
18	2b	Poland	f	*S. cinerea*	Shrub to 4m
18	3	Poland	f	*S. purpurea* × *S. viminalis*	
18	5	Poland	f	*S. triandra*	
18	6	Poland	f	*S. purpurea* × *S. viminalis*	
19	1	Poland	m	*S. ×fragilis*	Abundant at this site
19	2	Poland	f	*S. purpurea* × *S. viminalis*	
19	3	Poland	f	*S. viminalis*	
19	4	Poland	f	*S. triandra* × *S. viminalis*	
19	5	Poland	m	*S. alba*	Occasional at this site
19	6	Poland	f	*S. cinerea*	
20	1	Poland	m	*S. ×fragilis*	
20	2	Poland	f	*S. purpurea*	
20	3a	Poland	m	*S. triandra*	
20	3b	Poland	f	*S. triandra*	
20	4	Poland	f	*S. viminalis*	
20	5	Poland	f	*S. gmelinii*	
20a	1a	Poland	f	*S. triandra*	small tree/shrub to 4m
20a	1b	Poland	f	*S. triandra*	small tree/shrub to 4m
20a	2	Poland	f	*S. ×fragilis*	to 40m
20a	3	Poland	f	*S. alba*	to 30m
20a	4	Poland	f	*S. ×fragilis*	
20a	5	Poland	v	*S. gmelinii*	large open sprawling shrubs to 5m
20a	6	Poland	v	*S. viminalis*	shrub to 5m
20a	7	Poland	v	*S. purpurea*	shrub to 4m
20a	8	Poland	v	*S. triandra*	
20a	9	Poland	v	*S. ×fragilis*	very glossy green upper sides to leaves
21	1a	Poland	m	*S. triandra*	small trees or multi-stemmed shrubs to 10m high ×20m across
21	1b	Poland	f	*S. triandra*	
21	2	Poland	f	*S. ×fragilis*	small bush to large tree to 20m
21	3	Poland	v	*S. ×fragilis*	
21	4	Poland	f	*S. purpurea*	shrub to 4m
21	5	Poland	f	*S. cinerea*	
21	5a	Poland	f	*S. cinerea* × *S. triandra*	shrub to 3m
21	5b	Poland	v	*S. purpurea*	
21	6	Poland	f	*S. viminalis*	bush to 6m
21	7	Poland	f	*S. alba*	tree to 30m
21	8	Poland	f	*S. ×fragilis*	
21	9	Poland	f	*S. triandra*	
21	10	Poland	v	*S. purpurea*	
22	1	Poland	f	*S. ×fragilis*	15m medium tree
22	2	Poland	v	*S. bebbiana*	low bushes 2-3m with abundant cercopid spittle bugs and willow feeding scaraboid beetle
22	3	Poland	v	*S. ×fragilis*	shrub to 3m
22	4	Poland	v	*S. ×fragilis*	sapling 1.5m
22	5	Poland	v	*S. bebbiana*	
23	1	Poland	v	*S. bebbiana*	blue/grey-green foliage, 2-5m high
23	2	Poland	f	*S. pentandra*	trees all multistemmed (c. 4), to 15m, rugged bark, foliage with somewhat weeping habit
23	3	Poland	v	*S. myrsinifolia*	a few low bushes in the meadow with yellow-green foliage and some red pigmentation on stems, 1-1.5m
23	4	Poland	v	*S. bebbiana*	
23	5	Poland	v	*S. bebbiana*	
24	1	Lithuania	v	*S. caprea*	large shrub to 10m, planted?
24	2	Lithuania	v	*S. purpurea*	bushes to 2m
24	3	Lithuania	v	*S. viminalis* × *S. cinerea*	small sapling, no striae
24	4	Lithuania	f	*S. ×fragilis*	medium tree to 20m
24	5	Lithuania	v	*S. viminalis*	
24	6	Lithuania	v	*S. purpurea*	
24	7	Lithuania	m	*S. triandra*	
24	8	Lithuania	f	*S. triandra*	
24	9	Lithuania	f	*S. ×fragilis*	
24	12	Lithuania	f	*S. alba*	
25	1	Lithuania	v	*S. purpurea*	
25	2	Lithuania	f	*S. alba*	
25	3a	Lithuania	m	*S. pentandra*	
25	3b	Lithuania	f	*S. pentandra*	
25	4	Lithuania	f	*S. triandra*	
25	5	Lithuania	v	*S. myrsinifolia*	
25	6	Lithuania	v	*S. myrsinifolia*	
25	7	Lithuania	v	*S. myrsinifolia*	
25	8	Lithuania	v	*S. myrsinifolia*	
25	9	Lithuania	f	*S. cinerea*	
25	10	Lithuania	v	*S. triandra*	
25	11	Lithuania	v	*S. ×fragilis*	
26	1	Latvia	v	*S. cinerea*	5m h ×6m w
26	2	Latvia	m	*S. triandra*	
26	3	Latvia	m	*S. pentandra*	small trees to 8m
26	4	Latvia	v	*S. bebbiana*	
26	5	Latvia	v	*S. purpurea × viminalis*	
26	6	Latvia	v	*S. bebbiana*	
26	7	Latvia	v	*S. viminalis*	
26	8	Latvia	v	*S. myrsinifolia*	
26	9	Latvia	f	*S. myrsinifolia*	
26	10	Latvia	v	*S. caprea*	
26	11	Latvia	v	*S. alba*	
26	12	Latvia	v	*S. purpurea* × *S. viminalis*	
26	13	Latvia	f	*S. triandra*	
26	14	Latvia	f	*S. triandra*	
27	1	Latvia	f	*S. myrsinifolia*	subglabrous shrubs to 5m
27	2	Latvia	v	*S. bebbiana*	small bush with striae
27	3	Latvia	v	*S. viminalis*	
27	4	Latvia	f	*S. triandra*	
27	5	Latvia	v	*S. ×fragilis*	
28	1a	Estonia	m	*S. triandra*	wide bush, 3m h ×4m w
28	1b	Estonia	f	*S. triandra*	
28	2	Estonia	v	*S. ×fragilis*	young plants to 4m
28	3	Estonia	v	*S. ×fragilis*	
28	4	Estonia	f	*S. cinerea* × *S. aurita*	bush 3 ×4m, with striae
28	5	Estonia	v	*S. caprea*	vigorous bush to 4m, no striae
28	6	Estonia	f	*S. myrsinifolia*	bush to 2m
28	7	Estonia	f	*S. myrsinifolia*	
28	8	Estonia	v	*S. phylicifolia*	
28	9	Estonia	v	*S. myrsinifolia*	
29	1	Estonia	v	*S. purpurea* × *S. viminalis*	
29	2	Estonia	v	*S. ×fragilis* × *S. triandra*	
29	3	Estonia	f	*S. myrsinifolia*	
29	4	Estonia	v	*S. myrsinifolia*	
29	5	Estonia	v	*S. phylicifolia* × *S. myrsinifolia*	
29	6	Estonia	f	*S. myrsinifolia*	
30	1a	Finland	b	*S. phylicifolia*	shrub to 4m
30	1b	Finland	f	*S. phylicifolia*	
30	2	Finland	f	*S. pentandra*	
30	3	Finland	v	*S. aurita*	shrub to 4m, weakly striate
30	4	Finland	v	*S. caprea*	tree straight-trunked to 15m
30	5a	Finland	b	*S. phylicifolia*	
30	5b	Finland	f	*S. phylicifolia*	
30	6	Finland	v	*S. cinerea*	large shrub, no striae
30	7a	Finland	b	*S. phylicifolia*	
30	7b	Finland	v	*S. phylicifolia*	
31	1	Finland	f	*S. myrsinifolia*	small shrub to 2m
31	2	Finland	v	*S. phylicifolia* × *S. myrsinifolia*	
31	3	Finland	f	*S. myrsinifolia*	
31	4	Finland	v	*S. phylicifolia*	
31	5	Finland	v	*S. euxina* × *S. pentandra*	very small plants 1-2m
31	6	Finland	v	*S. myrsinifolia*	
31	7	Finland	f	*S. cinerea*	
31	8	Finland	f	*S. myrsinifolia*	small plants 1-2m
31	9	Finland	f	*S. myrsinifolia*	
31	10	Finland	f	*S. myrsinifolia*	
31	11	Finland	m	*S. pentandra*	
32	1	Finland	f	*S. pentandra*	to 4m
32	2	Finland	v	*S. aurita*	to 2m
32	3	Finland	f	*S. myrsinifolia*	to 2m
32	4	Finland	f	*S. phylicifolia*	to 2m
32	5	Finland	f	*S. myrsinifolia*	to 4m
32	6	Finland	f	*S. phylicifolia*	to 3m
32	7	Finland	f	*S. myrsinifolia*	
32	8	Finland	f	*S. myrsinifolia*	
32	9	Finland	v	*S. bebbiana*	
32	10	Finland	v	*S. caprea*	
33	1	Finland	v	*S. caprea*	small shrubs to 3m
33	2	Finland	f	*S. aurita*	to 2m
33	3	Finland	f	*S. myrsinifolia*	to 4m
33	4	Finland	f	*S. phylicifolia*	to 3m
34	1	Finland	f	*S. phylicifolia*	to 3m
34	2	Finland	v	*S. caprea*	to 3m
34	3	Finland	m	*S. pentandra*	c. 4m
34	4	Finland	f	*S. phylicifolia*	
34	5	Finland	f	*S. phylicifolia*	
35	1	Finland	v	*S. caprea*	small tree to 4m
35	2	Finland	v	*S. aurita* × S. *myrsinifolia*	shrub to 1.5m
35	3	Finland	f	*S. aurita*	old tree, 8m high
35	4	Finland	f	*S. phylicifolia*	bushes to 6m
35	5	Finland	f	*S. phylicifolia*	
35	6	Finland	f	*S. phylicifolia*	
35	7	Finland	f	*S. phylicifolia*	
36	1	Finland	f	*S. phylicifolia*	to 4m
36	2	Finland	f	*S. myrsinifolia*	to 6m
36	3	Finland	f	*S. phylicifolia*	
37	1	Finland	f	*S. phylicifolia*	shrub 1-3m
37	2	Finland	m	*S. hastata*	5m high spindly tree
37	3	Finland	v	*S. caprea*	3-4m high
37	4a	Finland	m	*S. hastata*	in wet heathy scrub, less than 75cm
37	4b	Finland	f	*S. hastata*	
37	5	Finland	f	*S. myrsinifolia*	
38	1	Finland	f	*S. phylicifolia*	to 3m
38	2	Finland	f	*S. glauca*	to 1.5m
38	3	Finland	f	*S. glauca*	
38	4	Finland	f	*S. hastata*	1-1.5m
38	5	Finland	m	*S. phylicifolia*	
38	6	Finland	v	*S. caprea*	
38	7	Finland	f	*S. hastata*	
38	8	Finland	f	*S. myrtilloides* × *S. glauca*	
38	9	Finland	v	*S. caprea*	
38	10	Finland	f	*S. lapponum*	
39	1	Finland	f	*S. phylicifolia*	bushes to 2m
39	2	Finland	f	*S. hastata*	shrub to 1.5m
39	3	Finland	f	*S. hastata*	
39	4	Finland	f	*S. phylicifolia*	
39	5	Finland	f	*S. bebbiana*	
39	6	Finland	f	*S. hastata*	
39	7	Finland	m	*S. phylicifolia*	
39	8	Finland	m	*S. hastata*	
39	9	Finland	m	*S. hastata*	
39	10	Finland	v	*S. caprea*	
39	11	Finland	v	*S. caprea*	
39	12	Finland	f	*S. glauca*	
39	13a	Finland	m	*S. glauca*	
39	13b	Finland	f	*S. glauca*	
39	14	Finland	f	*S. lapponum*	
39	15	Finland	f	*S. lapponum*	
40	1a	Norway	m	*S. phylicifolia*	
40	1b	Norway	f	*S. phylicifolia*	
40	2	Norway	b	*S. glauca*	
40	4	Norway	m/f	*S. phylicifolia*	catkins bisexual
40	5	Norway	f	*S. phylicifolia*	
40	6	Norway	f	*S. phylicifolia*	
41	1	Norway	f	*S. lapponum*	grey bush willow 1-1.5m
41	2	Norway	b	*S. glauca*	
41	3	Norway	f	*S. glauca*	
41	4	Norway	f	*S. phylicifolia*	green bush willow 1-2m
41	5	Norway	f	*S. phylicifolia*	
41	6	Norway	v	*S. hastata*	dwarf willow
41	7	Norway	v	*S. glauca*	
41	8	Norway	f	*S. myrsinifolia*	
41	9	Norway	f	*S. myrsinifolia*	5m high with slender dark grey stems
41	10	Norway	m	*S. myrsinifolia*	
41	11	Norway	f	*S. bebbiana*	
41	12	Norway	v	*S. caprea*	5m high with slender pale grey stems
42	1a	Norway	v	*S. lapponum*	
42	1b	Norway	f	*S. lapponum*	shrub 1-1.5m
42	2	Norway	m	*S. glauca*	shrub c. 1m
42	3a	Norway	m	*S. glauca*	dwarf shrub
42	3b	Norway	f	*S. lanata*	dwarf shrub
42	4	Norway	v	*S. caprea*	to 5m
42	5	Norway	f	*S. lanata*	shrub less than 75cm
42	6a	Norway	m	*S. myrsinifolia*	shrub to 4m
42	6b	Norway	f	*S. phylicifolia*	
42	7	Norway	f	*S. myrsinifolia*	shrub 5m
42	9a	Norway	m	*S. myrsinifolia*	
42	9b	Norway	f	*S. myrsinifolia*	
42	10	Norway	f	*S. hastata*	
42	11	Norway	f	*S. hastata*	
42	13	Norway	f	*S. lapponum*	
42	14	Norway	f	*S. glauca*	
42	15	Norway	f	*S. glauca*	
A-i	1	Switzerland	f	*S. eleagnos*	
A-i	2	Switzerland	m	*S. eleagnos*	
A-i	3	Switzerland	f	*S. purpurea* × *S. viminalis*	
A-1	4	Switzerland	m	*S. purpurea* × *S. viminalis*	
C-i	1	Greece	m	*S. alba*	
J-i	1	Romania	-	*S. cinerea* [Not collected]	
J-i	2	Romania	-	*S. fragilis* [Not collected]	
K-i	1	Holland	f	*S. caprea*	
C-ii	1	Estonia	f	*S. euxina* × *S. pentandra*	
C-ii	2	Estonia	v	*S. ×fragilis*	
C-ii	3	Estonia	v	*S. ×fragilis* × *S. triandra*	
C-ii	4	Estonia	b	*S. phylicifolia*	
C-ii	5	Estonia	v	*S. myrsinifolia*	
C-ii	6	Estonia	f	*S. cinerea*	
C-ii	7	Estonia	f	*S. cinerea*	
C-ii	8	Estonia	v	*S. bebbiana*	
C-ii	9	Estonia	f	*S. bebbiana*	
C-ii	10	Estonia	f	*S. phylicifolia*	
C-ii	11	Estonia	f	*S. myrsinifolia*	
C-ii	12	Estonia	f	*S. myrsinifolia*	
D	1	Finland	f	*S. aurita* × *S. cinerea*	
D	2	Finland	v	*S. caprea*	
D	3	Finland	f	*S. myrsinifolia* × *S. phylicifolia*	
E	1a	Finland	m	*S. lapponum*	
E	1b	Finland	f	*S. lapponum*	
E	2a	Finland	m	*S. bebbiana*	
E	2b	Finland	f	*S. bebbiana*	
H	1	Norway	m	*S. hastata*	
H	2	Norway	m	*S. hastata*	
H	3	Norway	m	*S. hastata*	
H	4	Norway	f	*S. phylicifolia*	
H	5	Norway	f	*S. phylicifolia*	
H	6	Norway	f	*S. myrsinifolia*	
H	7	Norway	f	*S. hastata*	
H	8	Norway	f	*S. phylicifolia*	
H	9	Norway	m	*S. glauca*	
H	10	Norway	f	*S. phylicifolia*	
H	11	Norway	f	*S. hastata*	
H	12	Norway	f	*S. glauca*	
H	13	Norway	f	*S. glauca*	
H	14	Norway	f	*S. lapponum*	
H	15	Norway	f	*S. glauca*	
H	16	Norway	f	*S. phylicifolia*	
I	1a	Norway	m	*S. myrsinifolia*	
I	1b	Norway	f	*S. caprea*	
I	1c	Norway	v	*S. caprea*	
I	2a	Norway	f	*S. myrsinifolia*	
I	2b	Norway	f	*S. myrsinifolia*	
I	2c	Norway	f	*S. myrsinifolia*	
I	2d	Norway	f	*S. myrsinifolia*	
I	2e	Norway	f	*S. myrsinifolia*	
I	3a	Norway	f	*S. hastat* a	
I	3b	Norway	f	*S. hastata*	
I	3c	Norway	f	*S. hastata*	
I	3d	Norway	f	*S. hastata*	
I	3e	Norway	f	*S. hastata*	
